# Diversity of prokaryotic microorganisms in alkaline saline soil of the Qarhan Salt Lake area in the Qinghai–Tibet Plateau

**DOI:** 10.1038/s41598-022-07311-3

**Published:** 2022-03-01

**Authors:** Yaqiong Wang, Guoyuan Bao

**Affiliations:** 1 School of Ecology, Environment and Resources, Qinghai Minzu University, Bayi Road, Xining, 810007 Qinghai China; 2 Qinghai Provincial Key Laboratory of High-Value Utilization of Characteristic Economic Plants, Xining, 810007 China; 3 Qinghai Provincial Biotechnology and Analytical Test Key Laboratory, Tibetan Plateau Juema Research Centre, Xining, 810007 China

**Keywords:** Microbiology, Ecology

## Abstract

The composition of microbial communities varies considerably across ecological environments, particularly in extreme environments, where unique microorganisms are typically used as the indicators of environmental conditions. However, the ecological reasons for the differences in microbial communities remain largely unknown. Herein, we analyzed taxonomic and functional community profiles via high-throughput sequencing to determine the alkaline saline soil bacterial and archaeal communities in the Qarhan Salt Lake area in the Qinghai–Tibet Plateau. The results showed that Betaproteobacteria (Proteobacteria) and Halobacteria (Euryarchaeota) were the most abundant in the soils of this area, which are common in high salinity environments. Accordingly, microbes that can adapt to local extremes typically have unique metabolic pathways and functions, such as chemoheterotrophy, aerobic chemoheterotrophy, nitrogen fixation, ureolysis, nitrate reduction, fermentation, dark hydrogen oxidation, and methanogenesis. Methanogenesis pathways include hydrogenotrophic methanogenesis, CO_2_ reduction with H_2_, and formate methanogenesis. Thus, prokaryotic microorganisms in high salinity environments are indispensable in nitrogen and carbon cycling via particular metabolic pathways.

## Introduction

Extreme environments are defined as harsh conditions that are uninhabitable for most living organisms^1^. They are characterized by environmental conditions such as pH, temperature, pressure, nutrients, or saline concentrations that are exceptionally high or low^2^. Extremophilic microorganisms, such as Thermophiles, Psychrophiles, Halophiles, Acidophiles, Alkalophiles, Anaerobe, Piezophiles, and Polyextremophiles, live in extreme environments because they have unique enzymatic systems, cellular structures, unique amino acid composition, or metabolic mechanisms^3–5^. Thus, extreme environments provide a unique opportunity to assess microbial types and complement our understanding of microbial growth parameters and requirements^1^.

As a result of new technologies such as fluorescence in situ hybridization (FISH) and metagenomics, life can now be detected in the most extreme environments. Among these, the study of microorganisms in hypersaline environments such as solar salterns^6,7^, saltpans^8^, salt mines^9^, oceans^5,10^, and salt lakes^11–16^ has received considerable interest. Microorganisms that have adapted to life at high salt concentrations are found in three domains of life: Archaea, Bacteria, and Eukarya^4,13,17–22^. The aerobic halophilic Archaea of the order Halobacteriales, family Halobacteriaceae, are the halophiles par excellence^23,24^. A few species of the methanogenic Archaea have adapted to life at high salt concentrations, the majority of which belong to the family Methanosarcinaceae^24^. Cultivation-independent metagenomic studies of hypersaline biota have recently led to the discovery of the third group of halophilic Archaea: the “Nanohaloarchaea^24,25^.” Many different types of halophilic and halotolerant microorganisms are found in the domain Bacteria, which is divided into many phylogenetic subgroups^26–29^. Halophiles are scarce within the domain Eucarya; however, the green alga *Dunaliella* is almost always present in high-salt environments^17,29^.

The polyextremophilic behavior of halophilic microorganisms makes them particularly useful in bioremediation processes^30^, biotechnological applications^31^, and as a potentially good choice in carbon, nitrogen, and sulfur cycling^32–37^. There is very little information on halophilic microorganisms in the inland Plateau salt lake region, and more research is required.

The Qarhan Salt Lake, located in the northeastern Qinghai–Tibet Plateau, is the largest in China, consisting of ten modern salt lakes^38^, with a total area of 5856 km^2^. The Qarhan Salt Lake area has an extremely arid desert climate; the mean annual temperature is 5.33 °C, mean annual precipitation is approximately 24 mm, annual evaporation is approximately 3564 mm, average wind speed is 4.3 m/s, and relative moisture is 27.7%^39^. Qarhan Salt Lake is also the largest large-scale inland comprehensive salt deposit in China with industrial exploitation value of quaternary stone salt, potassium salt, magnesium salt, and high concentrations of boron, lithium, rubidium, cesium, bromine, iodine, and other valuable chemical elements. The salt lake is primarily composed of potassium and magnesium brine ores that coexist with solid and liquid. Approximately 90% of the sodium chloride has been deposited into stable solid mineral layers. In contrast, the remainder of the potassium, magnesium, lithium, boron, rubidium, cesium, and other minerals are primarily found in brine^39^. The Qarhan Salt Lake is an important resource for both industry and agriculture, and it has been studied for decades. Researchers are also interested in the microbes in this area because they are representative of an extreme high-salt environment. Zhu et al*.*^40^ studied the core bacterial communities associated with hypersaline environments in lake water and sediments from the Qaidam Basin. Liu et al*.*^41^ investigated Gammaproteobacterial diversity and carbon utilization in lakes on the Qinghai–Tibet Plateau in response to salinity, while Zhong et al.^42^ studied the prokaryotic community structure influenced by salinity and ionic concentrations in plateau lakes of the Tibetan Plateau. However, there have been few studies on soil microorganisms in bare land and plant-covered saline-alkali land around salt lakes in this area, which merits further investigations.

Herein, we present a study of the prokaryotic community of hypersaline soil in the Qarhan Salt Lake area using high-throughput sequencing and the ecological function of prokaryotes in this area. This study aims to (1) improve our current understanding of the prokaryotic community in a previously uncharacterized inland hypersaline environment and (2) provide clues about how microbes adapt to the extreme environments of high salinity at high altitudes.

## Methods

### Sample collection

The sampling site is near the Qarhan Salt Lake (36°36′57″N, 95°11′24″E; altitude 2651 m) in the state of Qinghai–Tibet Plateau, China. Soil samples were collected during the summer, on July 14, 2020, at a temperature of 22 °C. Field experiment photographs are shown in Supplementary Fig. S1.

Five soil samples were collected; one from bare land (QSB) and the other four from the grassland (QSG1, QSG2, QSG3, and QSG4), with a distance of 100 m between each sample. Five sub-samples (100 g) were collected with a hand spade from the 0 to 10 cm layer, pooled, homogeneously mixed into one 500 g sample, and transported to the laboratory. The samples were sieved with a 5 mm test sieve (WSTYLER, USA) under aseptic conditions. A portion of the soil (250 g) was used to characterize soil properties, and the remainder (250 g) was stored at − 80 °C for sequencing. The contents of various elements in the soil samples were determined using the ZSX Primus IV X-ray fluorescence spectrometer (Rigaku, Japan) according to the manufacturer's instructions, and the results are summarized in Supplementary Table S1.

### DNA extraction, PCR, and sequencing

Total genomic DNA (gDNA) from soil samples (~ 500 mg) was extracted using the E.Z.N.A™ Mag-Bind Soil DNA Kit according to the manufacturer’s instructions (OMEGA Bio-Tek, USA). The DNA yield was quantified with the Qubit3.0 DNA Test Kit (Life Technologies, USA). Purified DNA was used as the template for the amplification of 16S rDNA genes via polymerase chain reaction (PCR). Approximately 10–20 ng of gDNA was used as a PCR template for amplification.

There were two rounds of nested PCR amplification for archaea. For the first round, the reaction mixture (30 µL) contained 10–20 ng of gDNA, the appropriate primers at 1 µL each, and 2 × Hieff® Robust PCR Master Mix (Yeasen Biotechnology, Shanghai, China) of 15 µL. The archaeal-specific primers used were GU1ST-340F (5′-CCCTAYGGGGYGCASCAG-3′) and GU1ST-1000R (5′-GGCCATGCACYWCYTCTC-3′)^43^. Amplification conditions included a denaturation step for 3 min at 94 °C, followed by 5 cycles consisting of 30 s at 94 °C, 20 s at 45 °C, 30 s at 65 °C; 20 cycles consisting of 20 s at 94 °C, 20 s at 55 °C, 30 s at 72 °C, and a final elongation step at 72 °C for 5 min. The 20–30 ng of the PCR product in the first round was used as template DNA for the second PCR, which was conducted using the same PCR conditions and general V3-V4 primer set 349F (5′-GYGCASCAGKCGMGAAW-3′), 806R (5′-GGACTACVSGGGTATCTAAT-3′), including a barcode on the forward primer.

For bacteria, the primers Nobar_341F (5′-CCTACGGGNGGCWGCAG-3′) and Nobar_805R (5′-GACTACHVGGGTATCTAATCC-3′)^43^ were used in the PCR, including a barcode on the forward primer. The PCR reactions were performed in 30 µL reactions for denaturation at 94 °C for 3 min, followed by 5 cycles consisting of 30 s at 94 °C, 20 s at 45 °C, 30 s at 65 °C; 20 cycles consisting of 20 s at 94 °C, 20 s at 55 °C, 30 s at 72 °C, and a final elongation step at 72 °C for 5 min.

Subsequently, Illumina bridge PCR compatible primers were introduced, and PCR was performed in 30 µL reactions containing 20–30 ng of PCR product of bacteria or archaea, which was used as template DNA, the primer F 1 µL, Index-PCR Primer R 1 µL, and 2 × Hieff® Robust PCR Master Mix (Yeasen) 15 µL. The PCR reactions included denaturation at 95 °C for 3 min, followed by 5 cycles of denaturation at 94 °C for 20 s, annealing at 55 °C for 20 s, extension at 72 °C for 30 s, and a final elongation step at 72 °C for 5 min.

PCR products were assessed via agarose gel electrophoresis. To obtain a uniform long cluster effect and high-quality sequencing data, the library concentration was determined using a Qubit 3.0 fluorometer (Invitrogen, USA). Subsequently, the amplicons were loaded onto an Illumina HiSeq platform (Illumina, Inc. San Diego, CA, USA), according to the manufacturer’s guidelines.

### Bioinformatics and statistical analyses

Sequences were analyzed using a combination of USEARCH 11.0.667 and QIIME v1.8.0^44^. The sequencing primer connector of the Read 3' -end was removed from Cutadapt 1.18^45^. PEAR 0.9.8 was used to merge the pairs of reads into a sequence according to the overlapping relationship between paired-end reads (PE reads)^46^. Barcodes were removed from the multiplexed FASTQ files using the USEARCH python command script fastq_strip_barcode_relabel2.py. PRINSEQ 0.20.4 was used to remove the bases with a tail mass value below 20 reads, and a window of 10 bp was set. If the average mass value in the window was lower than 20, the back-end bases were cut off to filter the N-containing sequences and short sequences after quality control, and the low-complexity sequences were finally filtered out^47^. The FASTA files were de-replicated, abundance sorted, and singleton sequences were removed. The operational taxonomic units (OTUs) were clustered de novo using USEARCH 11.0.667^48^. The OTUs were then mapped back to the original reads, and an OTU table was produced. Taxonomy was assigned to OTUs using the BLAST method in QIIME and against the RDP 16 S database 2.12: http://rdp.cme.msu.edu/misc/resources.jsp. Mothur 1.43.0 was used to determine the alpha diversity index^49^. Principal component analysis was used to reflect the differences and distances between samples using the vegan R package (v. 2.5-6).The relative abundances of bacterial taxa were summarized using the Venn diagram package (v. 1.6.20) for R^50^.

OTU co-occurrence network analysis was conducted using the R graph package (v. 2.0.0) based on the Bray–Curtis distance metric. Redundancy analysis (RDA) was conducted to evaluate the association between community composition and environmental parameters using the RDA function of the vegan package for R (v.2.5-6)^51^. Correlation heat maps were used evaluate the correlation between microbial classification and environmental variables using R (v.3.3.0).

The functional potential of the microbial community was investigated using 16S rRNA abundance data via PICRUSt v.1.1.4 with default parameters^52^. The 16S rRNA-based metagenome was functionally annotated using KEGG pathway functions using hidden state prediction^53^. The functional annotation of prokaryotic taxa via Functional Annotation of Prokaryotic Taxa (FAPROTAX) v.1.2.1 is available online (http://www.zoology.ubc.ca/louca/FAPROTAX)^54^.

## Results

### Microbial community structure in soils around salt lakes

Microbial community composition was investigated via high-throughput Illumina sequencing. The number of bacterial and archaeal sequences in the five samples were 205,563 and 283,308, respectively. A total of 643 OTUs were recovered, comprising 611 and 32 bacterial and archaeal OTUs, respectively. The rarefaction curves of all samples were flat, indicating that the amount of sequencing data was sufficient (See Supplementary Fig. S2).

**Figure 1:**
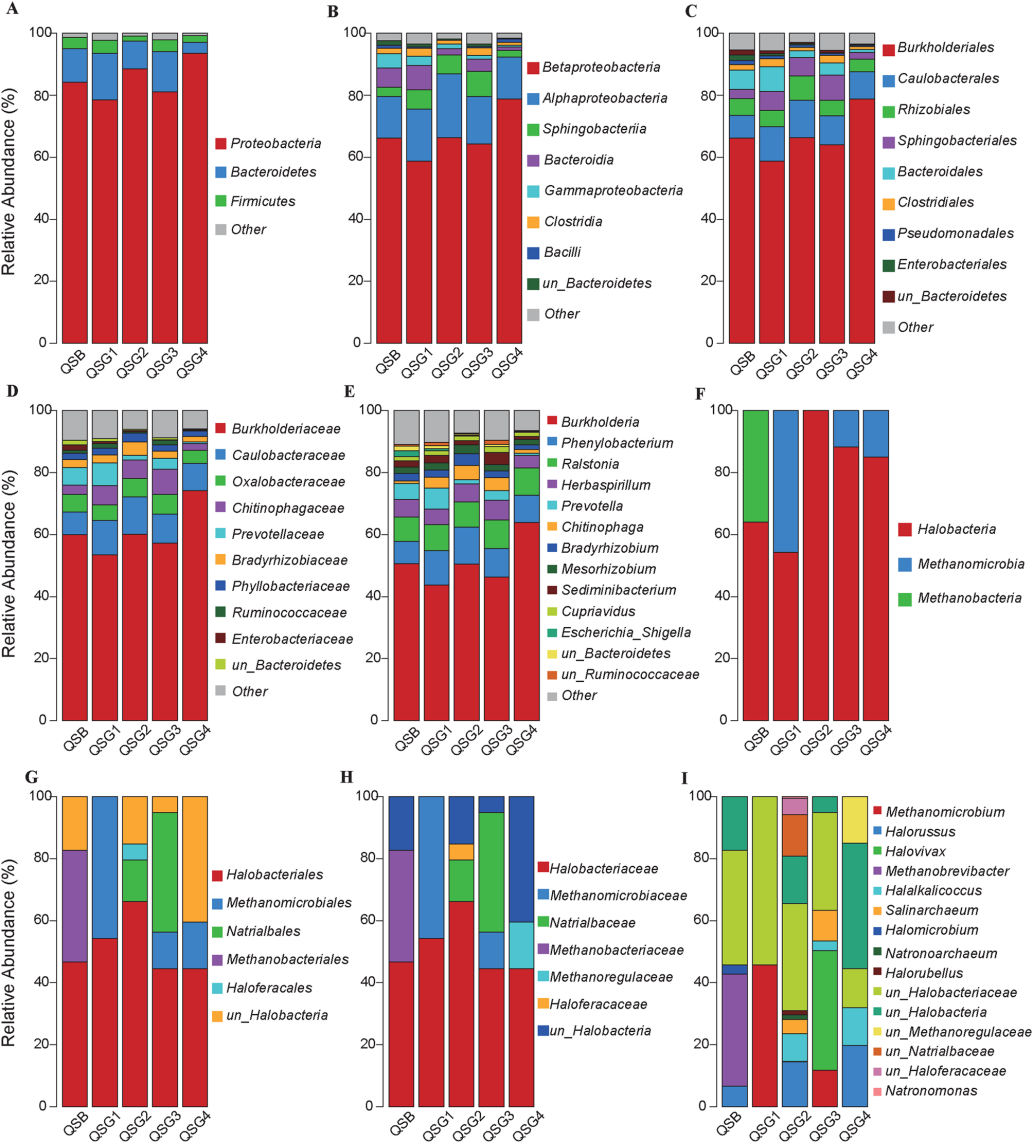
Relative abundance of prokaryotic microorganisms at different taxonomic units in soils around the Chaerhan Salt Lake. (**A**) Bacteria Phylum, (**B**) Bacteria Class, (**C**) Bacteria Order, (**D**) Bacteria Family, (**E**) Bacteria Genus, (**F**) Archaea Class, (**G**) Archaea Order, (**H**) Archaea Family, and (**I**) Archaea Genus). Groups occupying less than 1% of the distribution were clubbed together and designated as “Others”.

The bacterial domain was divided into 18 phyla, 42 classes, 66 orders, 115 families, and 195 genera. The dominant bacterial phyla (relative abundance > 10%) in the five samples belonged to Proteobacteria (85.08%), followed by Bacteroidetes (10.37%) and Firmicutes (2.99%); these three bacterial phyla constituted more than 98% of all reads (Fig. 1A). The major classes were Betaproteobacteria (66.65%), Alphaproteobacteria (16.01%), Sphingobacteriia (5.17%), Bacteroidia (4.24%), and Gammaproteobacteria (2.18%), which were among the top five of the total bacterial classes (Fig. 1B). At the order level, Burkholderiales (66.56%) were the most dominant, followed by Caulobacterales (9.75%), Rhizobiales (5.61%), Sphingobacteriales (5.17%), and Bacteroidales (4.24%) in total abundance (Fig. 1C). At the family level, Burkholderiaceae (60.76%) was dominant among all bacterial families (Fig. 1D). Several genera were frequently dominant, with proportions in total sequences of more than 1% (Fig. 1E). Among the dominant genera, *Burkholderia* was the most abundant (1 OTU, 50.77% of total sequences), followed by *Phenylobacterium* (1 OTU, 9.64%), *Ralstonia* (2 OTUs, 8.47%), *Herbaspirillum* (1 OTU, 5.43%), *Prevotella* (80 OTUs, 3.41%), *Chitinophaga* (1 OTU, 2.92%), *Bradyrhizobium* (1 OTU, 2.49%), *Mesorhizobium* (1 OTU, 2.17%), *Sediminibacterium* (1 OTU, 2.16%), and *Cupriavidus* (1 OTU, 1.52%) (Fig. 1E). These ten dominant genera accounted for 88.98% of the total classified sequences.

All the archaea detected belonged to the phylum Euryarchaeota, including 3 classes, 6 orders, 7 families, and 15 genera. Among these 3 classes, Halobacteria was the most abundant, accounting for 90.63% of the total 32 OTUs, covering 223,081 sequences (78.74% of total 283,308 reads), followed by Methanomicrobia (2 OTUs, 40,511 sequences [14.30%]) and Methanobacteria (1 OTU, 19,716 sequences [6.96%], Fig. 1F). Halobacteriales (51.30%) dominated among all bacterial orders (Fig. 1G), and Halobacteriaceae (51.30%) dominated among all bacterial families (Fig. 1H). At the genus level, the dominant archaeal genera (relative abundance > 10%) were *unclassified_Halobacteriaceae*, *unclassified_Halobacteria*, and *Methanomicrobium*, each with a widely varying abundance. The subdominant genera (1–10% relative abundance) consisted of *Halorussus*, *Halovivax*, *Methanobrevibacter*, *Halalkalicoccus*, unclassified_Methanoregulaceae, *Salinarchaeum*, unclassified_Natrialbaceae, and unclassified_Haloferacaceae. Other minor genera included *Halomicrobium*, *Natronoarchaeum*, *Halorubellus*, and *Natronomonas*, which constituted small percentages of community abundance (< 1%). (Fig. 1I).

Alpha diversity analysis revealed that bacterial and archaeal community richness (Chao1), diversity (Shannon and Simpson), and evenness (Shannoneven) varied widely among the samples (Table 1). In particular, the lowest bacterial richness, diversity, and evenness were samples from QSG4, and the highest richness was QSB, with the highest diversity and evenness being QSG1. For archaea, the lowest richness and diversity were samples from QSG1, and the highest were samples from QSG2; the lowest evenness was QSB, and the highest was QSG1.

**Table 1:** Statistical analysis of microbial diversity in the soil around the Qarhan Salt Lake on the Qinghai–Tibet Plateau.

Classification	Sample	Sequence number	OTUs	Chao	Shannon	Simpson	Coverage	Shannoneven
Bacteria	QSB	42,785	283	284.909	2.535	0.273	0.99984	0.449
	QSG1	39,352	266	266.857	2.690	0.216	0.99990	0.482
	QSG2	44,574	161	161.000	2.133	0.284	0.99998	0.420
	QSG3	41,922	272	272.500	2.529	0.239	0.99995	0.451
	QSG4	36,930	117	117.250	1.661	0.427	0.99995	0.349
Archaea	QSB	54,738	10	10.000	1.647	0.240	1.00000	0.715
	QSG1	54,344	2	2.000	0.690	0.504	1.00000	0.995
	QSG2	58,870	20	20.000	2.570	0.095	0.99998	0.858
	QSG3	52,600	6	6.000	1.473	0.275	1.00000	0.822
	QSG4	62,756	7	7.000	1.798	0.176	1.00000	0.924

### Alkaline saline soil prokaryotic β-diversity

**Figure 2:**
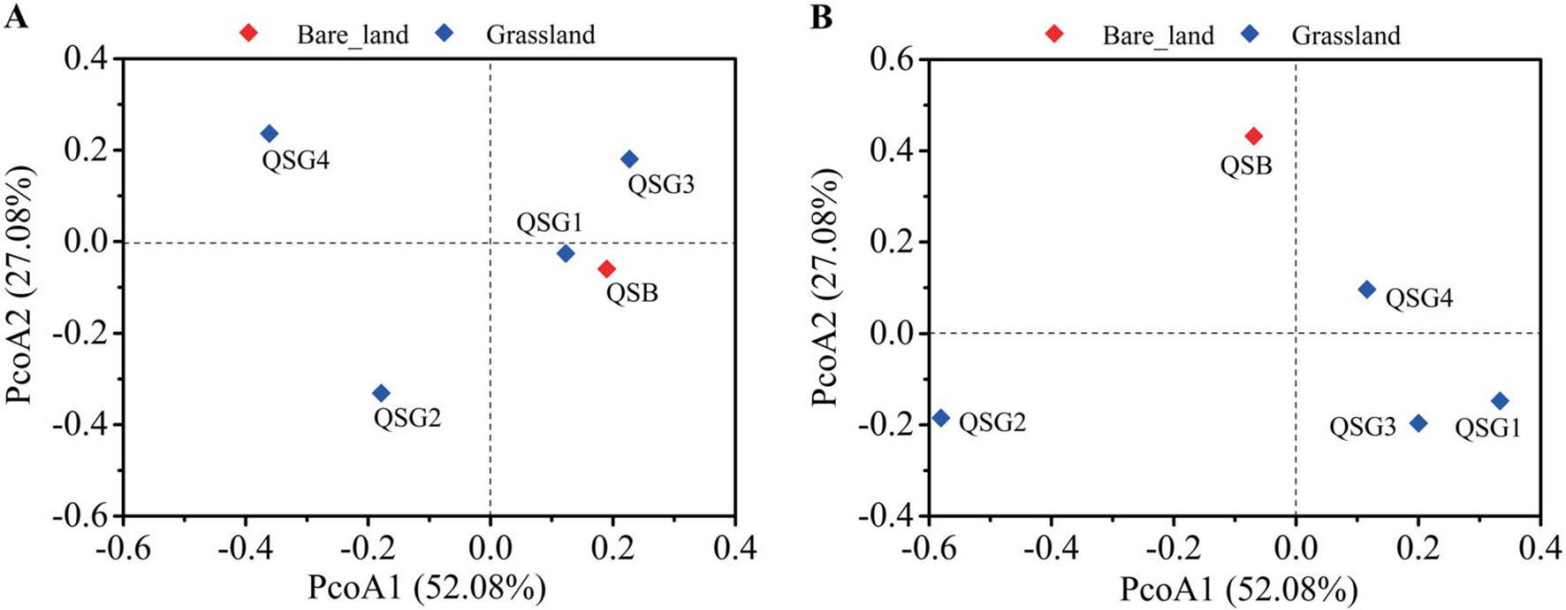
Principal coordinate (Unweighted Unifrac) plot showing the β-diversity of bacterial (**A**) and archaeal (**B**) communities in soils around the Chaerhan Salt Lake.

Unweighted UniFrac distance metrics were used to estimate bacterial and archaeal β-diversity and identify dissimilarities between the samples. The principal coordinate analysis (PCoA) plot illustrated the dissimilarity of OTU composition; the first two principal components explained 79.18% (PCoA 1 + PCoA 2; bacteria) and 79.18% (PCoA 1 + PCoA 2; archaea) of the total variations (Fig. 2). For the analysis of multivariate homogeneity among groups, the analysis of similarities (ANOSIM) test was performed, and the results showed that there were no significant differences between the bare land and the grassland (*p* > 0.05).

**Figure 3:**
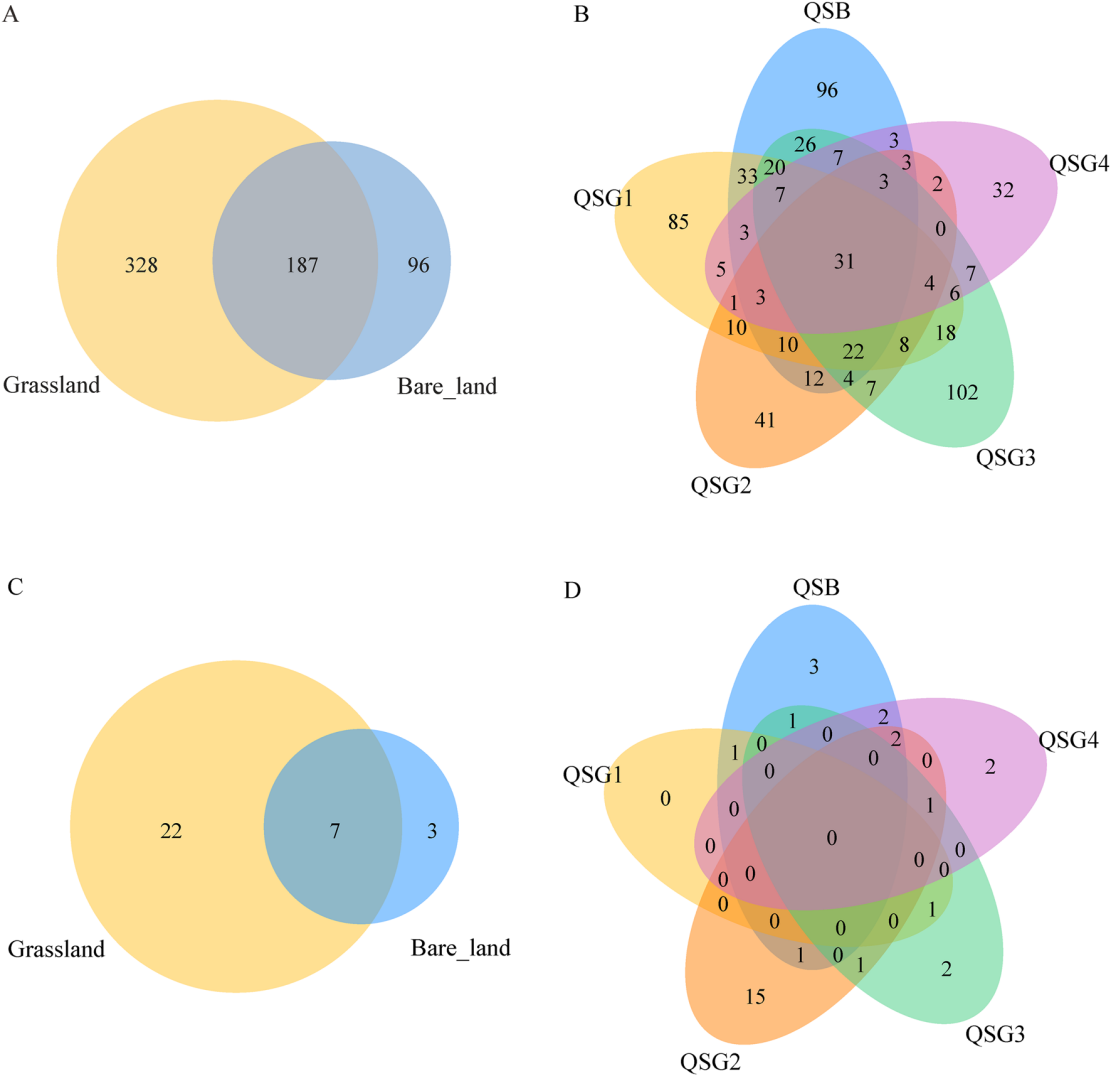
Venn diagram showing the number of shared and unique bacterial (**A**, **B**) and archaeal (**C**, **D**) OTUs in soils around the Chaerhan Salt Lake.

Bacteria from bare land and grassland shared 187 OTUs (Fig. 3A), and unique OTUs (102) were recovered from QSG3, a number that exceeded the unique OTUs found in bare land QSB (96) (Fig. 3B). For archaea, bare land and grassland shared seven OTUs (Fig. 3C), more unique OTUs (15) were recovered from QSG2, a number that also exceeded the unique OTUs found in bare land QSB (3) (Fig. 3D).

### Potential correlations between microbial communities and soil variables

RDA was performed to reveal the relationship between microbial community structures and the soil variables. The first two RDA axes explained 60.38% and 64.8% of the bacterial and archaeal community variations, respectively (See Supplementary Fig. S3).

**Figure 4:**
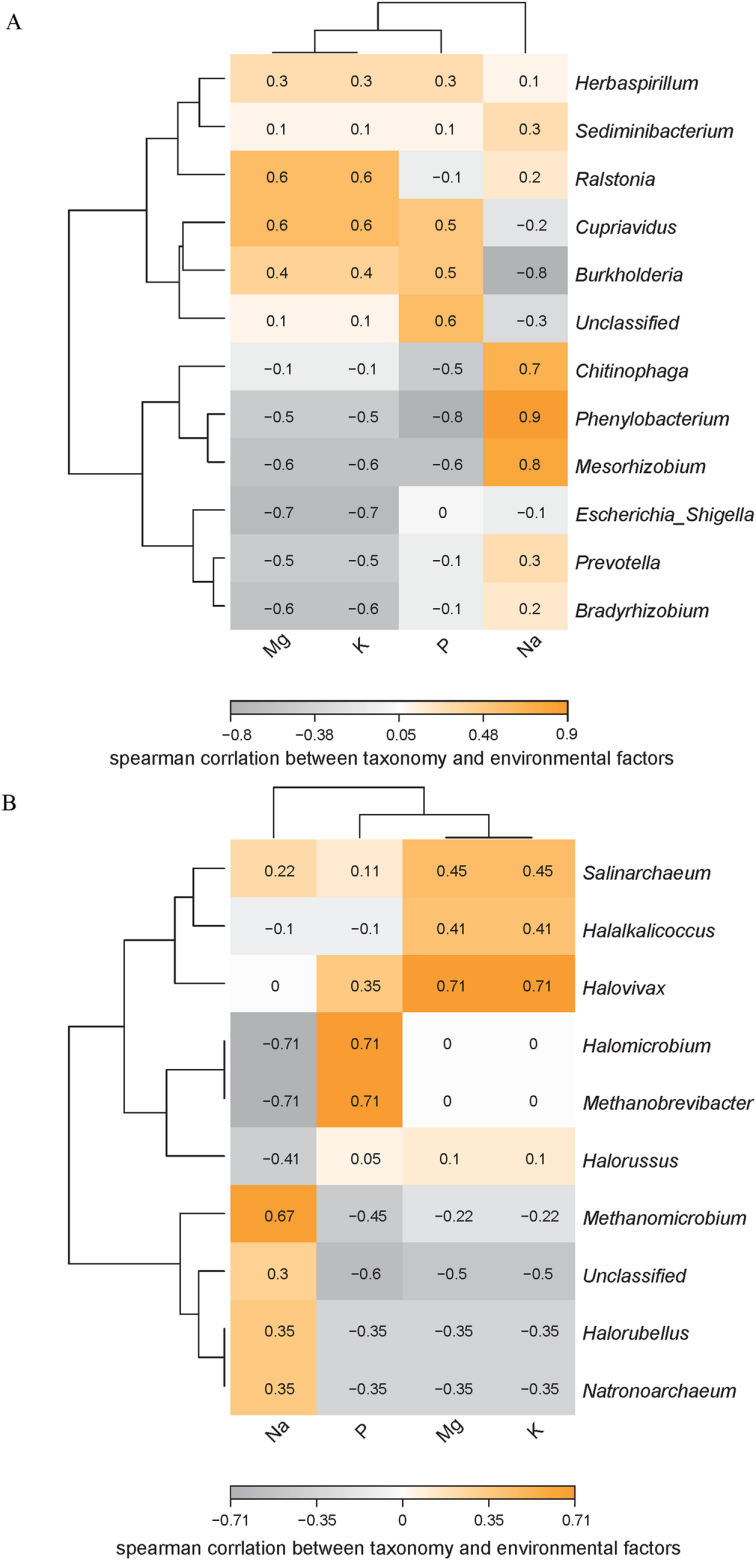
Heatmap of the bacterial (**A**) and archaeal (**B**) environment-sensitivity at the genus level in soils around the Chaerhan Salt Lake.

Spearman’s rank correlation test was performed to clarify the relationship between environmental factors and prokaryotic composition (relative abundance at the genus level) (Fig. 4). For bacteria, *Ralstonia* and *Cupriavidus* were positively correlated with Mg^2+^ and K^+^, whereas *Mesorhizobium*, *Escherichia, Shigella*, and *Bradyrhizobium* were negatively correlated with Mg^2+^ and K^+^; *Burkholderia* was negatively correlated with Na, whereas *Chitinophaga*, *Phenylobacterium,* and *Mesorhizobium* were positively correlated with the Na, and *Phenylobacterium* and *Mesorhizobium* were negatively correlated with P (Fig. 4A). For archaea, *Halovivax* was positively correlated with Mg^2+^ and K^+^; *Halomicrobium* and *Methanobrevibacter* were negatively correlated with Na but positively correlated with P; *Methanomicrobium* was positively correlated with Na (Fig. 4B). These findings suggest that soil variables are important contributing factors for the regulation of soil prokaryotes.

### Co‑occurrence network of dominant taxa among prokaryotic microorganisms

**Figure 5:**
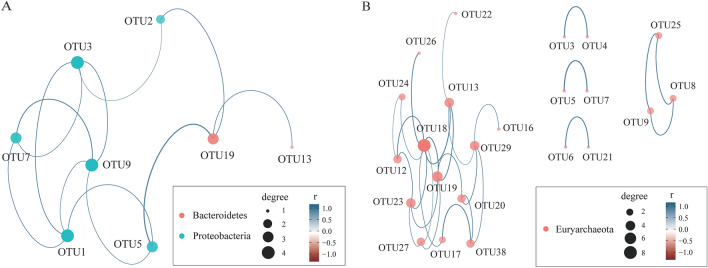
Bacterial (**A**) and archaeal (**B**) networks were constructed by calculating the correlations between species representing significant co-occurrence relationships among the abundance of clades on OTU level in soils around Chaerhan Salt Lake. The size of nodes in the figure represents the degree of connectivity of species, and different colors represent different gates. The colors of the lines indicate positive or negative correlations; the thickness of the line indicates the correlation coefficient, and the thicker the line, the higher the correlation between species. The more the lines, the closer the relationship between the species and other species. Only P-values < 0.05 and absolute values of correlation > 0.8 are shown in the figures.

A co‑occurrence network was constructed to identify the possible assemblages among prokaryotic microorganism OTUs in alkaline saline soil. The dominant core taxa in the cluster were strongly correlated with each other (∣R∣ > 0.8, p < 0.05). Notably, the network depicted several keystone OTUs that were assigned to the phyla Bacteroidetes, genus *Prevotella* (OTU19, and OTU13), Proteobacteria (OTU9—*Cupriavidus*, OTU3—*Ralstonia*, OTU1—*Burkholderia*, OTU5—*Mesorhizobium*, OTU7—*Herbaspirillum*, and OTU2—*Phenylobacterium*) (Fig. 5A). For archaeal taxa, including Halobacteria (OTU20, OTU8, and OTU17), Halobacteriaceae (OTU18, OTU29, OTU12, OTU25, OTU7, and OTU4), Haloferacaceae (OTU19), Natrialbaceae (OTU38, OTU24, OTU16, and OTU26), Methanoregulaceae (OTU9), *Halorussus* (OTU13 and OTU5), *Halorubellus* (OTU23), *Salinarchaeum* (OTU27), *Halovivax* (OTU3), *Methanobrevibacter* (OTU6), *Halomicrobium* (OTU21), and *Natronoarchaeum* (OTU22) (Fig. 5B).

The co-occurrence network effectively reveals the relationship between individual group members and the entire ecosystem ^55,56^. The co-occurrence network clusters suggest that core bacterial and archaeal taxa in alkaline saline soil are likely to collaborate and play a role in key metabolic steps in response to environmental changes. (Fig. 5). Thus, the study of physiological and metabolic characteristics belonging to these key species can help us understand the mechanisms of microbial adaptation to the environment.

### Prediction of the ecological function of prokaryotic microorganisms

To gain insights into the ecological role of bacteria and archaea in alkaline saline soil, the prediction tools PICRUSt and FAPROTAX were used to determine the functional characteristics of the prokaryotic communities in the soil. Table 2 presents the number of sequence reads of the predicted genes involved in adaptation to a high-salt environment.

**Table 2:** Metabolic enzymes for which cellular abundance was related to adaptation to high-salt conditions.

Taxa	Enzyme No	KEGG No	Type of enzyme	Abundance				
				QSB	QSG1	QSG2	QSG3	QSG4
Bacteria	1.4.1.13/1.4.1.14	K00266	Glutamate synthase (NADPH/NADH) small chain	30,397	30,001	33,352	31,344	26,406
	6.3.1.2	K01915	Glutamine synthetase	26,337	26,850	31,833	27,808	23,459
	1.2.1.8	K00130	Betaine-aldehyde dehydrogenase	24,450	19,588	25,485	22,474	25,803
	1.5.3.1	K00303	Sarcosine oxidase, subunit beta	10,861	8942	12,185	10,113	10,499
	1.5.1.2	K00286	Pyrroline-5-carboxylate reductase	10,282	10,506	11,645	10,995	8691
	1.4.1.13/1.4.1.14	K00265	Glutamate synthase (NADPH/NADH) large chain	9881	10,661	11,912	11,643	7185
	2.7.7.42	K00982	Glutamate-ammonia-ligase adenylyltransferase	8380	7815	9945	8467	7853
	1.4.1.2	K00260	Glutamate dehydrogenase	6967	6655	8671	7069	6739
	1.4.1.3	K00261	Glutamate dehydrogenase (NAD(P)+)	7014	6237	7835	7698	6566
	1.5.3.1	K00302	Sarcosine oxidase, subunit alpha	6707	5610	7793	6285	6074
	1.5.3.1	K00304	Sarcosine oxidase, subunit delta	6678	5590	7723	6244	6078
	1.5.3.1	K00305	Sarcosine oxidase, subunit gamma	6224	5161	7112	5809	5746
	3.6.3.32	K02000	Glycine betaine/proline transport system ATP-binding protein	5905	4772	6228	5528	5424
	3.1.3.12	K01087	Trehalose-phosphatase	5640	4492	6090	5153	5230
	3.1.6.6	K01133	Choline-sulfatase	4959	4072	5275	4672	4815
	1.4.7.1	K00284	Glutamate synthase (ferredoxin)	3810	3187	4049	3581	4117
	1.5.3.1	K00301	Sarcosine oxidase	2319	2942	3882	2762	2178
	1.4.1.4	K00262	Glutamate dehydrogenase (NADP+)	2850	2865	1420	2223	869
	1.14.11.-	K00674	Ectoine hydroxylase	227	508	704	647	205
	3.2.1.93	K01226	Trehalose-6-phosphate hydrolase	150	96	48	35	5
	4.2.1.108	K06720	l-Ectoine synthase	107	38	37	71	64
	2.3.1.178	K06718	l-2,4-Diaminobutyric acid acetyltransferase	103	38	37	55	61
	1.5.3.1/1.5.3.7	K00306	Sarcosine oxidase/l-pipecolate oxidase	0	0	0	12	0
Archaea	1.4.1.3	K00261	Glutamate dehydrogenase (NAD(P)+)	35,517	44,521	82,617	112,244	57,820
	6.3.1.2	K01915	Glutamine synthetase	35,133	59,573	35,278	50,496	34,394
	1.4.1.13/1.4.1.14	K00265	Glutamate synthase (NADPH/NADH) large chain	26,151	19,646	62,308	53,274	49,897
	1.5.3.1	K00303	Sarcosine oxidase, subunit beta	20,100	9823	36,957	61,074	24,948
	1.5.1.2	K00286	Pyrroline-5-carboxylate reductase	20,592	34,698	17,267	14,484	17,282
	3.1.6.6	K01133	Choline-sulfatase	15,417	9823	20,790	31,252	17,282
	1.4.1.13/1.4.1.14	K00266	Glutamate synthase (NADPH/NADH) small chain	0	49,750	0	12,380	9446
	3.1.3.12	K01087	Trehalose-phosphatase	4683	0	14,914	29,822	7667
	1.2.1.8	K00130	Betaine-aldehyde dehydrogenase	4683	0	12,724	29,822	7667
	3.6.3.32	K02000	Glycine betaine/proline transport system ATP-binding protein	0	24,875	0	6190	4723
	1.5.3.1	K00301	Sarcosine oxidase	0	0	12,265	6864	7667
	1.4.1.4	K00262	Glutamate dehydrogenase (NADP+)	9858	0	0	0	0
	1.4.1.2	K00260	Glutamate dehydrogenase	0	0	459	0	256

**Figure 6:**
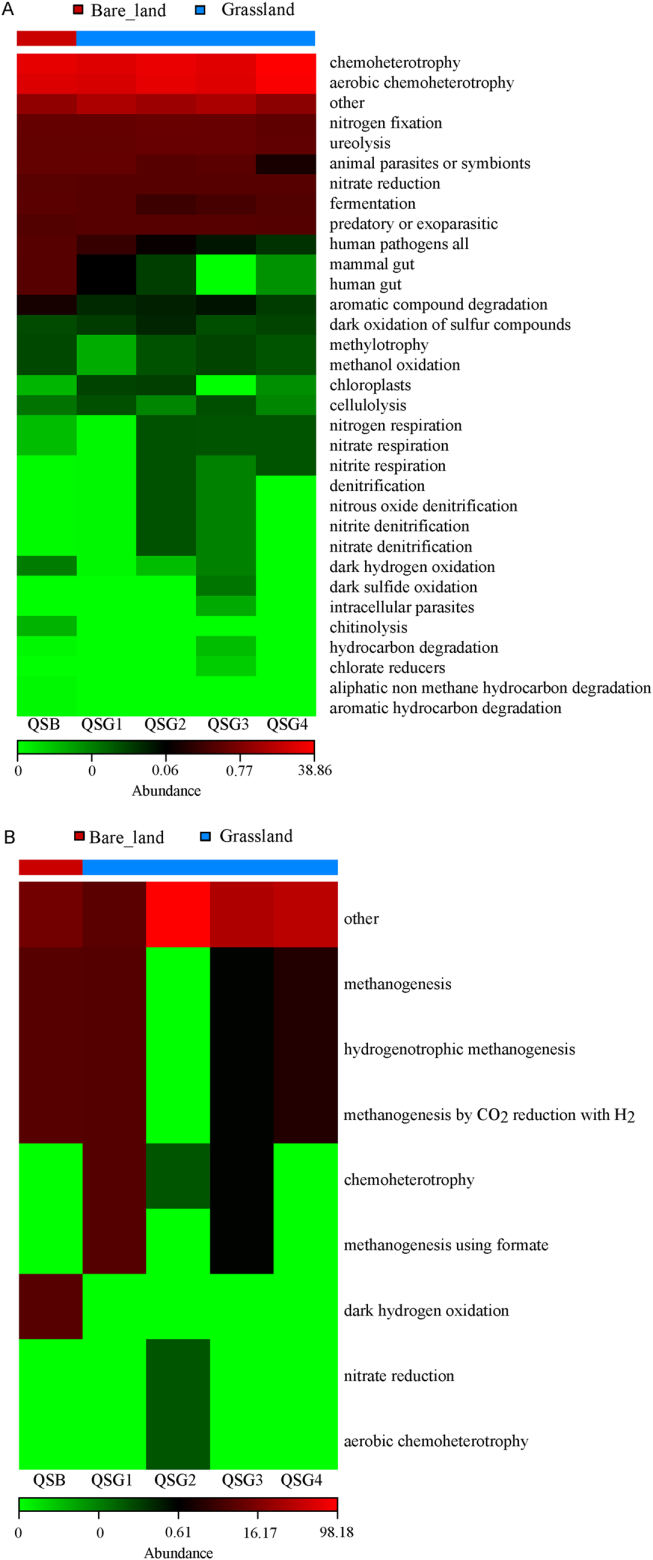
Functional community heatmap. Predict gene families based on prokaryotic metagenomes by modeling genes from 16S rRNA data derived from the generated OTUs and its reference genome database using FAPROTAX (**A**—bacteria and **B**—archaea). Red colors correspond to higher relative abundances.

The OTUs detected in all samples were compared with the FAPROTAX annotation rule in an automated manner; however, most OTUs could not be assigned to any functional group. Thus, only those OTUs that were successfully annotated were analyzed. Chemoheterotrophy, aerobic chemoheterotrophy, nitrogen fixation, ureolysis, nitrate reduction, fermentation, predatory, and exoparasitic were the most abundant bacterial functional groups (Fig. 6A). Methanogenesis, hydrogenotrophic methanogenesis, methanogenesis by CO_2_ reduction with H_2_, chemoheterotrophy, methanogenesis using formate, dark hydrogen oxidation, nitrate reduction, and aerobic chemoheterotrophy were the most abundance archaeal functional groups (Fig. 6B). These functional groups provide directions for understanding the mechanisms of the adaptation of prokaryotes to high salinity environments.

**Figure 7:**
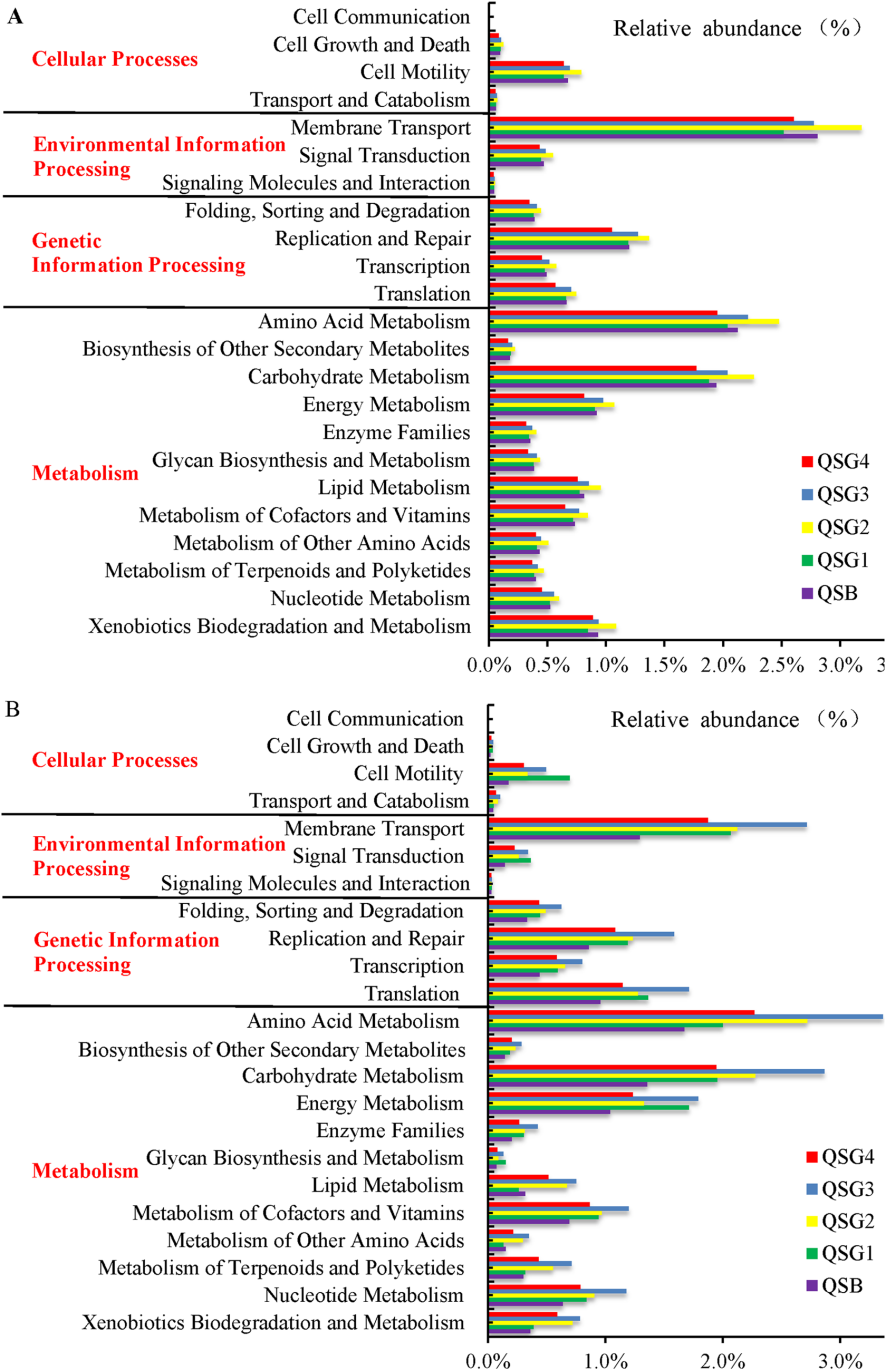
Relative abundances of metabolic pathways on KEGG categories (level 2) (**A**—bacteria and **B**—archaea).

The metabolic pathways of microbial consortia predicted by PICRUSt were further analyzed. Metabolic pathways were identified at three levels. The functions of bacteria and archaea related to the high-salt environment in level 1 include cellular processes (4.19–4.31%, 1.78–3.99%), environmental information processing (15.87–17.12%, 10.74–12.55%), genetic information processing (13.44–14.32%, 17.18–18.99%), and metabolism (49.29–49.62%, 46.69–52.02%). The distribution of bacterial and archaeal functions at level 2 was investigated further. For bacteria, the relative abundances of membrane transport, amino acid metabolism, carbohydrate metabolism, and replication and repair were enriched in the alkaline saline soil, with little difference between the samples (Fig. 7A). However, the relative abundances of amino acid metabolism, carbohydrate metabolism, membrane transport, energy metabolism, and translation for archaea were enriched in alkaline saline soil. There was a great deal of variations among samples (Fig. 7B). It is reasonable that bacteria and archaea may adopt different strategies when coping with extreme environments, and the bacterial community is relatively stable, while the archaea community is reasonably different.

## Discussion

Metagenomic technology is a powerful tool to explore microorganisms in extreme habitats and their environmental adaptation mechanisms^57^. Using this technique, we found that the predominant phyla within the bacterial communities were Proteobacteria (85.08%), followed by Bacteroidetes (10.37%), Firmicutes (2.99%), and Actinobacteria (0.34%), and Proteobacteria were ubiquitous across all samples in the soil of the Qarhan Salt Lake area (Fig. 1A). Numerous studies have revealed that the bacterial communities are dominated by Proteobacteria, followed by Firmicutes, Bacteroidetes, Cyanobacteria, Actinobacteria, and Verrucomicrobia^11,40,58^. Among the Proteobacteria, Alpha—(16.01%), Beta—(66.65%), Gamma—(2.18%), and Delta-Proteobacteria (0.18%) were detected in all samples (Fig. 1B). These taxa have previously been confirmed in other hypersaline environments^9,42,59,60^, which is consistent with the present results. Betaproteobacteria were dominant in the salt water and sediments from Chott El Jerid Lake (75%–95%)^11^, and other studies have revealed that Gammaproteobacteria^10,40,41^ and Alphaproteobacteria were the dominant classes^61^.

Bacteroidetes was the second most abundant phylum; it has been linked to nutrient conversion in lake sediments^62,63^. Its relative abundance in inland lakes is strongly correlated with the salinity gradient^42,64–66^. The network revealed two keystone OTUs assigned to the phyla Bacteroidetes, genus *Prevotella* (OTU19 and OTU13). Figure 5, combined with the abundance and widespread distribution, demonstrates its ecological significance in the alkaline saline soil.

The phyla Firmicutes and Actinobacteria were also found in hypersaline environments, which is consistent with our findings^11,67^. Actinobacteria can decompose cellulose and other organic materials in hypersaline environments^68^. Thus, the ecological role of Actinobacteria is particularly important in vegetation-covered saline-alkali land.

The top ten bacterial genera (> 1% of all sequences) accounted for 88.98% of the microbial community. *Burkholderia* was the most abundant, followed by *Phenylobacterium* and *Ralstonia* (Fig. 1E). Consistent with other studies^11^, *Burkholderia* predominated in our samples, and it has previously been reported to degrade aromatic hydrocarbons^69^. *Ralstonia* was also a common taxon in hypersaline environments^11,70,71^.

Archaea play an important role in the carbon and nitrogen^72^. The results showed that archaea in the soil near Qarhan Salt Lake were dominated by Halobacteria (Fig. 1F). Previous research has also revealed that Halobacteria live in salt lakes and salterns and propagate in salt crystals^9^.

The dominant family in these samples was Halobacteriaceae (51.30%) (Fig. 1H), which is consistent with studies of archaea from Ebinur Lake Wetland^73^, heavy metal-contaminated soils^74^, salt pans around Bhavnagar Coast^75^, inland saltern ecosystems in the Alto Vinalopó Valley^76^, and Lake Gasikule of the Tibetan Plateau^42^. These results showed that Halobacteriaceae was the dominant family in the majority of terrestrial high-salt environments. Halobacteriaceae can accumulate large quantities of inorganic ions (K^+^, Na^+^, and Cl^−^). Their intracellular proteins and macromolecules are not damaged by high intracellular salt concentrations^77^, ensuring their survival and dominance in high salt environments.

The genus-level composition of archaea varied greatly between samples (Fig. 1I). In particular, *Methanomicrobium* predominated in samples QSG1 and QSG3, whereas *Methanobrevibacter* predominated in sample QSB, which is uncommon in other related studies. Nevertheless, the core genus is significantly different from other high salt environments and represents a relatively unique archaea community. However, the percentage of unannotated archaea (56.96%) is remarkable.

Network interactions between taxa can capture ecological community habitat preference and taxa interactions^78,79^. Statistically, in our prokaryotic microorganisms in the alkaline saline soil of the Qarhan Salt Lake area, several keystone OTUs with high degrees were identified (Fig. 5), indicating that these OTUs could make a crucial difference in the soil microbial ecosystem. The metabolism of these keystone taxa is likely to be critical for the overall stability of the ecosystem, maintaining a fragile ecological balance in high-altitude and high-salt environments. Thus, the dynamics of any identified keystone OTUs may have a significant impact on this ecosystem.

The majority of the bacterial and archaeal species in the microbial community in the Qarhan Salt Lake area’s alkaline saline soil had genes involved in synthesizing halo-adaptation compounds such as ectoine, glycine betaine, glutamate, trehalose, and choline (Table 2). This result, similar to a study of bacterial communities in Lake Tuz, indicates that halophilic microbes' unique cellular enzymatic machinery enables them to effectively use hydrocarbons as their sole source of both carbon and energy^80^.

## Supplementary Information

Supplementary Information.

## References

[1] Boutaiba S, Hacene H, Bidle KA, Maupin-Furlow JA (2011). Microbial diversity of the hypersaline Sidi Ameur and Himalatt Salt Lakes of the Algerian Sahara. J. Arid Environ..

[2] Ventosa, A. Unusual micro-organisms from unusual habitats: hypersaline environments. *Symposia Society for General Microbiology* (2006).

[3] Fukuchi S, Yoshimune K, Wakayama M, Moriguchi M, Nishikawa K (2003). Unique amino acid composition of proteins in halophilic bacteria. J. Mol. Biol..

[4] Pillai SD, Nakatsu CH, Miller RV, Yates MV (2015). Manual of environmental microbiology. Life High-Salinity Environ..

[5] Poli A (2017). Microbial diversity in extreme marine habitats and their biomolecules. Microorganisms.

[6] Azpiazu-Muniozguren M, Martinez-Ballesteros I, Gamboa J, Seoane S, Bikandi J (2021). *Altererythrobacter muriae* sp. nov., isolated from hypersaline Aana Salt Valley spring water, a continental thalassohaline-type solar saltern. Int. J. Syst. Evol. Microbiol..

[7] Zhang J (2016). Bacterial diversity in Bohai Bay Solar Saltworks, China. Curr. Microbiol..

[8] Highfield A, Ward A, Pipe R, Schroeder DC (2021). Molecular and phylogenetic analysis reveals new diversity of *Dunaliella salina* from hypersaline environments. J. Mar. Biol. Assoc. UK.

[9] Cycil LM (2020). Metagenomic insights into the diversity of halophilic microorganisms indigenous to the Karak Salt Mine, Pakistan. Front. Microbiol..

[10] Jacob JH, Hussein EI, Shakhatreh MAK, Cornelison CT (2017). Microbial community analysis of the hypersaline water of the Dead Sea using high-throughput amplicon sequencing. Microbiol. Open.

[11] Ben Abdallah M (2018). Abundance and diversity of prokaryotes in ephemeral hypersaline lake Chott El Jerid using Illumina Miseq sequencing, DGGE and qPCR assay. Extremophiles.

[12] Tazi L, Breakwell DP, Harker AR, Crandall KA (2014). Life in extreme environments: Microbial diversity in Great Salt Lake, Utah. Extremophiles.

[13] Kashi FJ, Owlia P, Amoozegar MA, Kazemi B (2021). Halophilic prokaryotes in Urmia Salt Lake, a hypersaline environment in Iran. Curr. Microbiol..

[14] Sorokin DY, Roman P, Kolganova TV (2021). Halo(natrono)archaea from hypersaline lakes can utilize sulfoxides other than DMSO as electron acceptors for anaerobic respiration. Extremophiles.

[15] Hwang K, Choe H, Kim KM (2021). Complete genome of *Nocardioides aquaticus* KCTC 9944T isolated from meromictic and hypersaline Ekho Lake, Antarctica. Mar. Genom..

[16] Didari M (2020). Diversity of halophilic and halotolerant bacteria in the largest seasonal hypersaline lake (Aran-Bidgol-Iran). J. Environ. Health Sci. Eng..

[17] Oren A (2002). Diversity of halophilic microorganisms: Environments, phylogeny, physiology, and applications. J. Ind. Microbiol. Biotechnol..

[18] Mutlu MB (2008). Prokaryotic diversity in Tuz Lake, a hypersaline environment in Inland Turkey. FEMS Microbiol. Ecol..

[19] Antón J (2008). Distribution, abundance and diversity of the extremely halophilic bacterium Salinibacter ruber. Saline Syst..

[20] Oren A (2008). Microbial life at high salt concentrations: phylogenetic and metabolic diversity. Saline Syst..

[21] Abdeljabbar H, Badiaa E, Jean-Luc C, Marie-Laure F, Najla S (2014). Prokaryotic biodiversity of halophilic microorganisms isolated from Sehline Sebkha Salt Lake (Tunisia). Afr. J. Microbiol. Res..

[22] Najjari A, Elshahed MS, Cherif A, Youssef NH, Löffler FE (2015). Patterns and determinants of halophilic archaea (Class Halobacteria) diversity in Tunisian endorheic salt lakes and Sebkhet systems. Appl. Environ. Microbiol..

[23] Aharon O (1994). The ecology of the extremely halophilic archaea. FEMS Microbiol. Rev..

[24] Oren A (2019). Halophilic Archaea. FEMS Microbiol. Rev..

[25] Feng Y (2021). The evolutionary origins of extreme halophilic archaeal lineages. Genome Biol. Evol..

[26] Ventosa A, Nieto JJ, Oren A (1998). Biology of moderately halophilic aerobic bacteria. Microbiol. Mol. Biol. Rev..

[27] Kushner DJ (1968). Halophilic bacteria. Adv. Appl. Microbiol..

[28] Ghozlan H, Deif H, Kandil RA, Sabry S (2006). Biodiversity of moderately halophilic bacteria in hypersaline habitats in Egypt. J. Gen. Appl. Microbiol..

[29] Ali I, Prasongsuk S, Akbar A, Aslam M, Rakshit SK (2016). Hypersaline habitats and halophilic microorganisms. Maejo Int. J. Sci. Technol..

[30] Margesin R, Schinner F (2001). Biodegradation and bioremediation of hydrocarbons in extreme environments. Appl. Microbiol. Biotechnol..

[31] Poosarla VG, Ts C (2021). Xylanase production by halophilic bacterium *Gracilibacillus* sp. TSCPVG under solid state fermentation. Res. J. Biotechnol..

[32] Foti M (2007). Diversity, activity, and abundance of sulfate-reducing bacteria in saline and hypersaline soda lakes. Appl. Environ. Microbiol..

[33] Boujelben I (2012). Spatial and seasonal prokaryotic community dynamics in ponds of increasing salinity of Sfax solar saltern in Tunisia. Antonie Van Leeuwenhoek.

[34] García-Maldonado JQ, Bebout BM, Everroad RC, López-Cortés A (2014). Evidence of novel phylogenetic lineages of methanogenic archaea from hypersaline microbial mats. Microb. Ecol..

[35] Abed RMM, de Beer D, Stief P (2014). Functional-structural analysis of nitrogen-cycle bacteria in a hypersaline mat from the omani desert. Geomicrobiol. J..

[36] Coban O, Rasigraf O, Jong A, Spott O, Bebout BM (2021). Quantifying potential N turnover rates in hypersaline microbial mats by 15 N tracer techniques. Appl. Environ. Microbiol..

[37] Rodriguez-Valera F (1993). Introduction to Saline Environments.

[38] Wei, H. C., Qi-Shun, F., Fu-Yuan, A., Fa-Shou, S. & Qin, Z. J. Chemical elements in core sediments of the qarhan salt lake and palaeoclimate evolution during 94–9 ka. *Acta Geosci. Sin. *(2016).

[39] Yu S, Liu X, Tan H, Cao G (2009). Sustainable Utilization of Qarhan Salt Lake Resources.

[40] Zhu D (2020). An evaluation of the core bacterial communities associated with hypersaline environments in the Qaidam Basin, China. Arch. Microbiol..

[41] Liu W, Jiang H, Yang J, Wu G (2018). Gammaproteobacterial diversity and carbon utilization in response to salinity in the lakes on the qinghai-tibetan plateau. Geomicrobiol. J..

[42] Zhong Z-P (2016). Prokaryotic community structure driven by salinity and ionic concentrations in plateau lakes of the tibetan plateau. Appl. Environ. Microbiol..

[43] He C (2019). Synergistic effect of magnetite and zero-valent iron on anaerobic degradation and methanogenesis of phenol. Biores. Technol..

[44] Caporaso JG (2010). QIIME allows analysis of high-throughput community sequencing data. Nat. Methods.

[45] Martin M (2011). Cutadapt removes adapter sequences from high-throughput sequencing reads. Embnet J..

[46] Zhang J, Kassian K, Tomáš F, Alexandros S (2014). PEAR: a fast and accurate Illumina Paired-End reAd mergeR. Bioinformatics.

[47] Schmieder R, Edwards R (2011). Quality control and preprocessing of metagenomic datasets. Bioinformatics.

[48] Edgar RC (2013). UPARSE: Highly accurate OTU sequences from microbial amplicon reads. Nat. Methods.

[49] Schloss PD (2009). Introducing mothur: Open-source, platform-independent, community-supported software for describing and comparing microbial communities. Appl. Environ. Microbiol..

[50] Chen H, Boutros PC (2011). VennDiagram: A package for the generation of highly-customizable Venn and Euler diagrams in R. BMC Bioinform..

[51] McArdle BH (2001). Fitting multivariate models to community data: A comment on distance-based redundancy analysis. Ecology.

[52] Langille MGI (2013). Predictive functional profiling of microbial communities using 16S rRNA marker gene sequences. Nat. Biotechnol..

[53] Louca S, Doebeli M (2017). Efficient comparative phylogenetics on large trees. Bioinformatics.

[54] Louca S, Parfrey LW, Doebeli M (2016). Decoupling function and taxonomy in the global ocean microbiome. Science.

[55] Junker BH, Schreiber F (2008). Analysis of Biological Networks.

[56] Faust K, Raes J (2012). Microbial interactions: From networks to models. Nat. Rev. Microbiol..

[57] Behzad H, Ibarra MA, Mineta K, Gojobori T (2016). Metagenomic studies of the Red Sea. Gene.

[58] Naghoni A (2017). Microbial diversity in the hypersaline Lake Meyghan, Iran. Sci. Rep..

[59] Kambura AK (2016). Bacteria and Archaea diversity within the hot springs of Lake Magadi and Little Magadi in Kenya. BMC Microbiol..

[60] Paul D (2016). Exploration of microbial diversity and community structure of Lonar lake: The only hypersaline meteorite crater lake within basalt rock. Front. Microbiol..

[61] Ventosa A, de la Haba RR, Sánchez-Porro C, Papke RT (2015). Microbial diversity of hypersaline environments: A metagenomic approach. Curr. Opin. Microbiol..

[62] Liu FH (2009). Bacterial and archaeal assemblages in sediments of a large shallow freshwater lake, Lake Taihu, as revealed by denaturing gradient gel electrophoresis. J. Appl. Microbiol..

[63] Song H, Li Z, Du B, Wang G, Ding Y (2012). Bacterial communities in sediments of the shallow Lake Dongping in China. J. Appl. Microbiol..

[64] Wu QL, Zwart G, Schauer M, Agterveld KV, Hahn MW (2006). Bacterioplankton community composition along a salinity gradient of sixteen high-mountain lakes located on the Tibetan Plateau, China. Appl. Environ. Microbiol..

[65] Xing P, Hahn MW, Wu QL (2009). Low taxon richness of bacterioplankton in high-altitude lakes of the Eastern Tibetan Plateau, with a predominance of bacteroidetes and *Synechococcus* spp. Appl. Environ. Microbiol..

[66] Liu Y (2009). Bacterial diversity of freshwater Alpine Lake Puma Yumco on the Tibetan Plateau. Geomicrobiol. J..

[67] MounÃc S, Caumette P, Matheron R, Willison JC (2003). Molecular sequence analysis of prokaryotic diversity in the anoxic sediments underlying cyanobacterial mats of two hypersaline ponds in Mediterranean salterns. FEMS Microbiol. Ecol..

[68] Valenzuela-Encinas C (2009). Changes in the bacterial populations of the highly alkaline saline soil of the former lake Texcoco (Mexico) following flooding. Extremophiles.

[69] Kim TJ, Lee EY, Kim YJ, Cho K-S, Ryu HW (2003). Degradation of polyaromatic hydrocarbons by Burkholderia cepacia 2A–12. World J. Microbiol. Biotechnol..

[70] Gales G (2016). Preservation of ancestral *Cretaceous microflora* recovered from a hypersaline oil reservoir. Sci. Rep..

[71] Kleinsteuber S, Riis V, Fetzer I, Harms H, Müller S (2006). Population dynamics within a microbial consortium during growth on diesel fuel in saline environments. Appl. Environ. Microbiol..

[72] Valenzuela-Encinas C (2012). The archaeal diversity and population in a drained alkaline saline soil of the former lake Texcoco (Mexico). Geomicrobiol. J..

[73] He S, Tan J, Hu W, Mo C (2019). Diversity of archaea and its correlation with environmental factors in the Ebinur Lake Wetland. Curr. Microbiol..

[74] Sandaa RA, Enger O, Torsvik V (1999). Abundance and diversity of Archaea in heavy-metal-contaminated soils. Appl. Environ. Microbiol..

[75] Dave BP, Soni A (2012). Diversity of halophilic archaea at salt pans around Bhavnagar Coast, Gujarat. Proc. Natl. Acad. Sci. India B.

[76] Zafrilla B, Martínez-Espinosa R, Alonso MA, Bonete MJ (2010). Biodiversity of Archaea and floral of two inland saltern ecosystems in the Alto Vinalopó Valley, Spain. Saline Syst..

[77] Costa M, Santos H, Galinski EA (1998). An overview of the role and diversity of compatible solutes in Bacteria and Archaea. Adv. Biochem. Eng. Biotechnol..

[78] Williams RJ, Howe A, Hofmockel KS (2014). Demonstrating microbial co-occurrence pattern analyses within and between ecosystems. Front. Microbiol..

[79] Schmidt TSB, MatiasRodrigues JF, von Mering C (2016). A family of interaction-adjusted indices of community similarity. ISME J..

[80] Oyewusi HA (2020). Functional profiling of bacterial communities in Lake Tuz using 16S rRNA gene sequences. Biotechnol. Biotechnol. Equip..

