# Supplementary material for: Diversity of prokaryotic microorganisms in alkaline saline soil of the Qarhan Salt Lake area in the Qinghai–Tibet Plateau
Source: Sci Rep. 2022 Mar 1;12:3365. doi: 10.1038/s41598-022-07311-3 (PMC8888737; doi:10.1038/s41598-022-07311-3)
Supplement: Supplementary file 1 — Supplementary Information. [file 41598_2022_7311_MOESM1_ESM.pdf]

Supplementary Information

Diversity of prokaryotic microorganisms in alkaline saline soil of the Qarhan Salt Lake area in the Qinghai–Tibet Plateau

Yaqiong Wang[1,2,3*], Guoyuan Bao[1]

[1]School of Ecology, Environment and Resources, Qinghai Minzu University, Xining, 810007, China

[2]Qinghai Provincial Key Laboratory of High-value Utilization of Characteristic Economic Plants, Xining, 810007, China

[3]Qinghai Provincial Biotechnology and Analytical Test Key Laboratory, Tibetan Plateau Juema Research Centre, Xining, 810007, China

*Correspondence to: Yaqiong Wang

Qinghai Minzu University, Bayi Road, Xining, Qinghai 810007, China.

Telephone: 86-18297178818

Email: wangyaqiong727@163.com

Supplementary Figure S1. Qarhan Salt Lake (A, B); soil samples taken around Qarhan Salt Lake(C); salt particles precipitated from the soil in the sampling area(D).

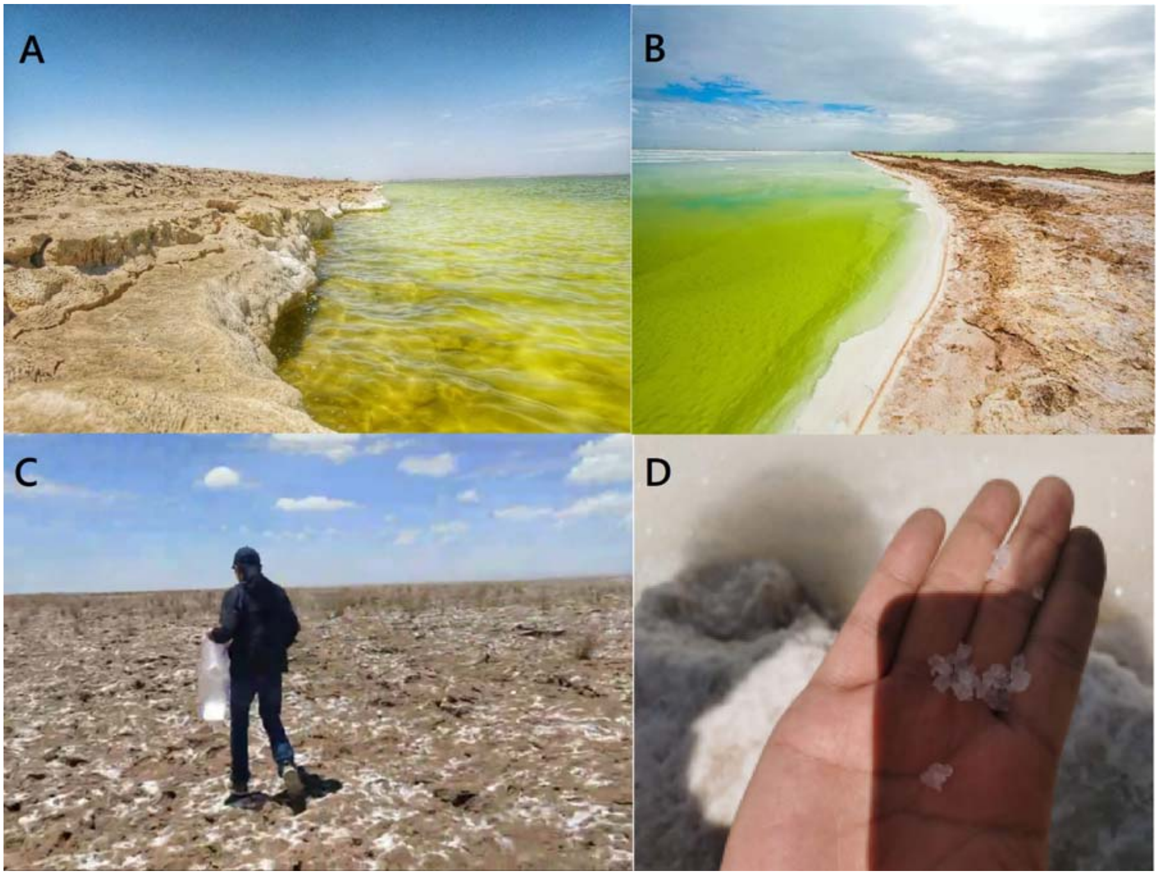

Supplementary Figure S2. Rarefaction curves depicting diversity of OTUs as a function of 16S rRNA gene sequencing effort. A corresponded to bacterial communities, while B corresponded to archaeal communities.

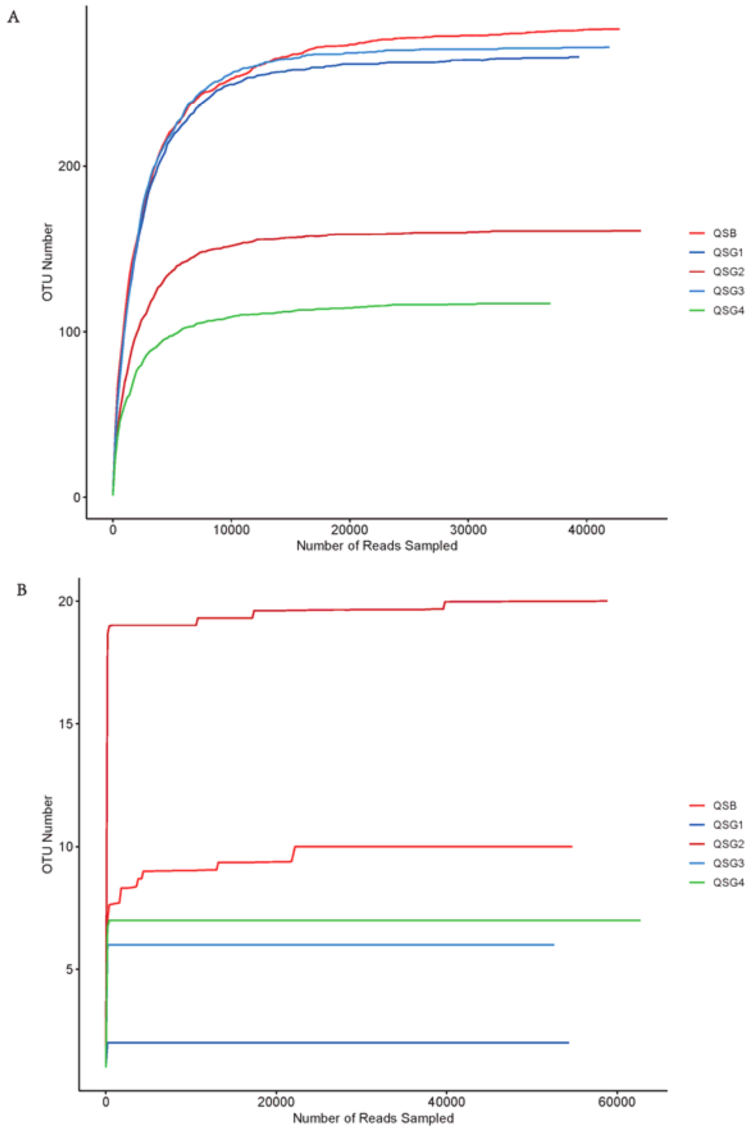

Supplementary Figure S3. Redundancy analysis of the selected soil environmental factor for the bacterial (A) and archaeal (B) community structures on OTU level in soils around the Chaerhan Salt Lake. When the included angle between environmental factors is acute, it means there is a positive correlation between the two environmental factors, and when it is obtuse, it means there is a negative correlation between the two environmental factors. The longer the arrow of the environmental factor is, the greater the influence of the factor is. The community structure of the sample projected the line of the arrow of the environmental factor. The closer the projection point was to the arrow, the greater the influence of the environmental factor on the community structure of the sample.

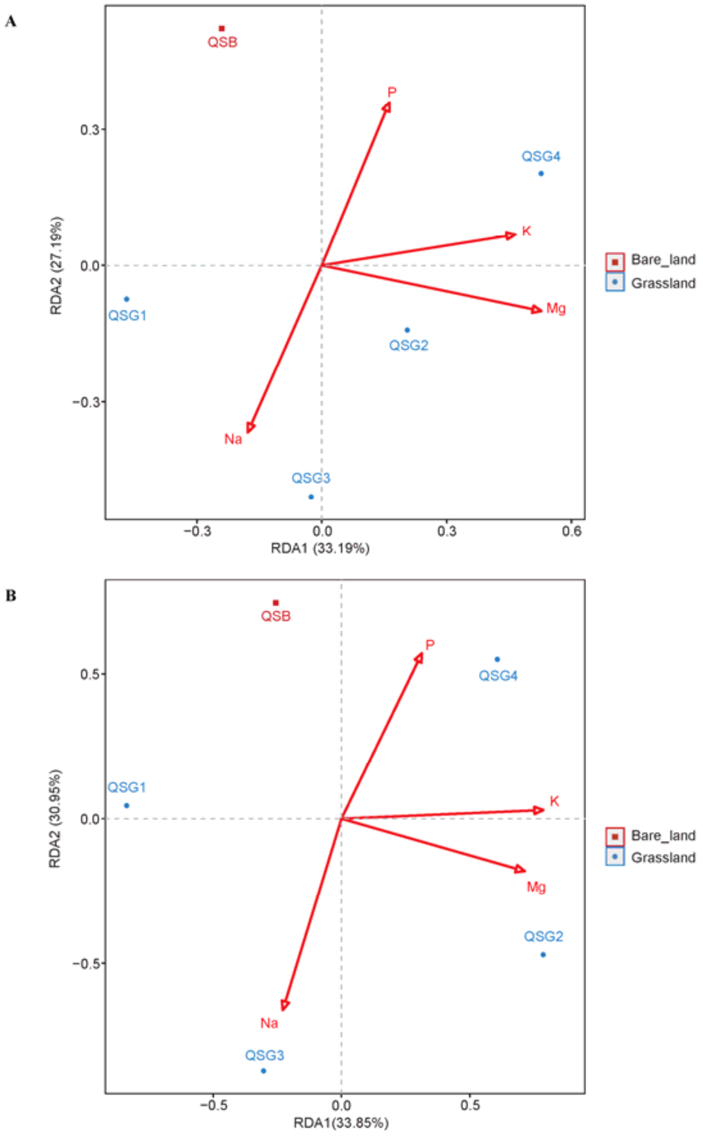

Supplementary Table S1. Characteristic analysis of environmental chemistry in the soil around the Qarhan Salt Lake on the Qinghai—Tibet plateau

Element content (%)	QSB	QSG1	QSG2	QSG3	QSG4
Na	11.1128	17.8679	16.1613	13.5551	13.433
Mg	1.6898	1.3046	1.6134	2.0121	2.0032
Al	5.0891	3.1556	4.1173	4.9606	4.7243
Si	19.4218	11.2012	15.5324	18.8318	18.0481
P	0.0866	0.0391	0.0564	0.0685	0.0664
S	10.3525	10.5251	8.8907	9.8433	12.192
Cl	26.3134	41.4844	37.8027	30.9194	29.186
K	2.7335	1.863	2.4804	2.8471	2.8056
Ca	15.3103	8.7217	8.3681	10.7735	11.6308
Ti	0.5106	0.2928	0.4149	0.5518	0.495
V	-	-	-	0.0184	-
Cr	0.0259	-	0.0228	0.0229	-
Mn	0.1156	0.0672	0.0816	0.1132	0.0989
Fe	5.7782	3.095	3.9977	4.9832	4.8193
Co	-	-	0.0026	-	-
Ni	0.0103	0.0102	0.0088	0.0101	0.0078
Cu	0.3492	0.0993	0.1134	0.1228	0.1
Zn	0.0456	0.0159	0.0192	0.021	0.0224
Se	0.3895	0.1117	0.1264	0.1365	0.0969
Rb	0.0186	0.0107	0.0136	0.0189	0.0169
Sr	0.2223	0.1222	0.0706	0.0894	0.1394
Y	0.0014	0.0012	0.0025	0.0017	0.0018
Zr	0.019	0.0112	0.023	0.0269	0.0233
Nb	0.2868	-	-	0.0024	-
Ba	0.0819	-	0.0801	0.0692	0.0836
W	0.0298	-	-	-	-
Pb	0.0058	-	-	-	0.0054
